# Activation and increase of radio-sensitive CD11b+ recruited Kupffer cells/macrophages in diet-induced steatohepatitis in FGF5 deficient mice

**DOI:** 10.1038/srep34466

**Published:** 2016-10-06

**Authors:** Hiroyuki Nakashima, Masahiro Nakashima, Manabu Kinoshita, Masami Ikarashi, Hiromi Miyazaki, Hiromi Hanaka, Junko Imaki, Shuhji Seki

**Affiliations:** 1 Department of Immunology and Microbiology National Defense Medical College, Namiki 3-2, Tokorozawa, Saitama 359-8513, Japan; 2 Department of Traumatology National Defense Medical College Research Institute, Namiki 3-2, Tokorozawa, Saitama 359-8513, Japan; 3 Department of Developmental Anatomy and Regenerative Biology National Defense Medical College, Namiki 3-2, Tokorozawa, Saitama 359-8513, Japan

## Abstract

We have recently reported that Kupffer cells consist of two subsets, radio-resistant resident CD68+ Kupffer cells and radio-sensitive recruited CD11b+ Kupffer cells/macrophages (Mφs). Non-alcoholic steatohepatitis (NASH) is characterized not only by hepatic steatosis but also chronic inflammation and fibrosis. In the present study, we investigated the immunological mechanism of diet-induced steatohepatitis in fibroblast growth factor 5 (FGF5) deficient mice. After consumption of a high fat diet (HFD) for 8 weeks, FGF5 null mice developed severe steatohepatitis and fibrosis resembling human NASH. F4/80+ Mφs which were both CD11b and CD68 positive accumulated in the liver. The production of TNF and FasL indicated that they are the pivotal effectors in this hepatitis. The weak phagocytic activity and lack of CRIg mRNA suggested that they were recruited Mφs. Intermittent exposure to 1 Gy irradiation markedly decreased these Mφs and dramatically inhibited liver inflammation without attenuating steatosis. However, depletion of the resident subset by clodronate liposome (c-lipo) treatment increased the Mφs and tended to exacerbate disease progression. Recruited CD11b+ CD68+ Kupffer cells/Mφs may play an essential role in steatohepatitis and fibrosis in FGF5 null mice fed with a HFD. Recruitment and activation of bone marrow derived Mφs is the key factor to develop steatohepatitis from simple steatosis.

Non-alcoholic steatohepatitis (NASH) is characterized by hepatic inflammation and fibrosis associated with steatosis, which in turn progresses to cirrhosis and hepato-cellular carcinoma^1^,^2^. In order to prevent the onset of this disease, it is important to elucidate a pathological mechanism for the induction of inflammation in simple liver steatosis. However, precise immunological mechanisms have not been revealed so far, even if the implication of innate immune cells, such as Kupffer cells, NKT cells and NK cells, has been discussed based on various experimental models^3^,^4^,^5^,^6^.

Recently, substantial progress has been made in the cellular physiology of macrophages (Mφs) and the existence of phenotypical, functional, and developmental differences in Mφ populations has been reported by some researchers^7^,^8^,^9^,^10^. Mφ populations, such as Kupffer cells in the liver, Langerhans cells in the skin, and microglia in the brain, have been demonstrated to differentiate from organ specific progenitor cells derived from the yolk sac, not from bone marrow derived monocytes^11^. Based on these pieces of research, the complex functions of Mφs in various pathophysiological conditions and diseases need to be reconsidered.

In previous studies, we reported that mouse Kupffer cells can be classified into two distinctive subsets, one of them resident type CD68+ cells derived from c-kit+ precursor cells in the liver, and the other CD11b+ Kupffer cells/ Mφs derived from bone marrow^12^,^13^. Surprisingly, the primary function of each population is entirely different; the former has a phagocytic and bactericidal function and the latter population plays a critical role in hepatic inflammation by secreting proinflammatory cytokines^13^, which may also be the case in Kupffer cells in humans^13^. We have also reported that excess intake of dietary cholesterol recruits and activates CD11b+ Kupffer cells/Mφs in the liver and severe hepatic injury is induced upon challenge with bacterial components (bacterial DNA motifs, CpG DNA)^6^. Based on our research and other reports dealing with human subjects, the CD11b population seems to play a pivotal role in the induction of inflammation and fibrosis in the pathogenesis of NASH^14^,^15^,^16^.

FGF5 is known to be a molecule that determines the length of body hair of mammals^17^,^18^,^19^ including humans^20^, and mice deficient in this gene have long hair (LH) compared to wild type (WT) mice^21^. Recently, some studies have reported that the single nucleotide mutation (polymorphism) of this gene is associated with hypertension in humans^22^,^23^,^24^,^25^. We have reported that a high fat diet (HFD) increased non-HDL cholesterol levels in FGF5 null LH mice and induced steatohepatitis resembling human NASH^21^. In the present study, based on our recent subclassification of Kupffer cell phenotypes, we investigated the immunological mechanism of this experimental model of NASH.

## Results

### HFD induce steatohepatitis and fibrosis in FGF5 null LH mice

**Figure 1:**
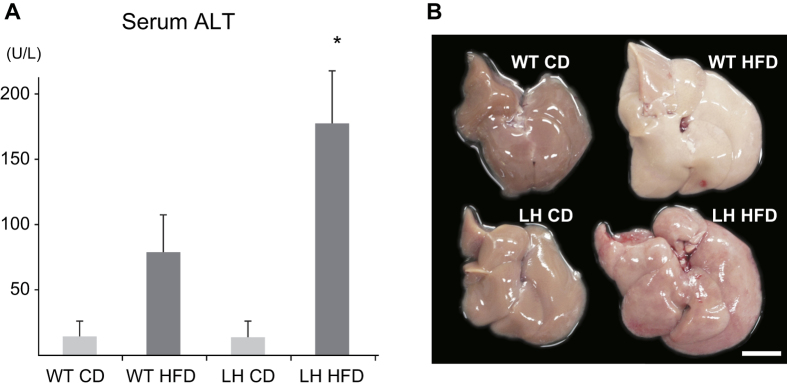
Serum ALT and liver macroscopic findings for FGF5 null or control mice. FGF5 null LH mice or WT ICR mice were fed HFD or CD for 8 weeks and sera and livers were collected. (**A**) Serum ALT levels. Data are the means ± SE from 10 mice in each group. *p < 0.05 vs. other groups. (**B**) Macroscopic findings for livers from mice of each group.

**Figure 2:**
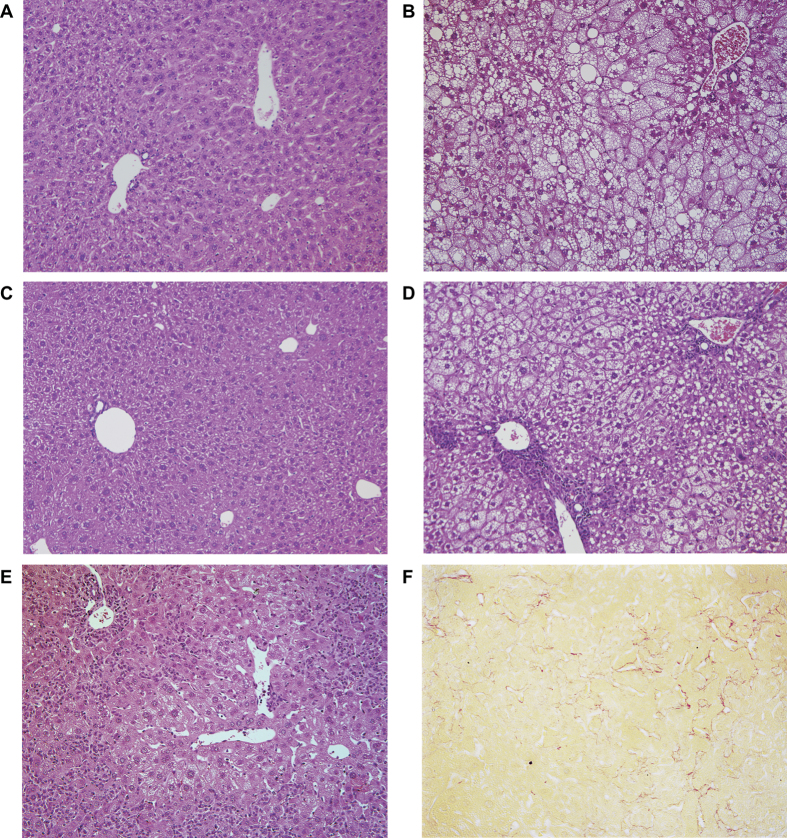
HE and Sirius red staining of FGF5 null or control mice livers. Collected livers were subjected to pathological analysis. (**A**) WT CD, (**B**) WT HFD, (**C**) LH CD, (**D**) LH HFD moderate liver injury, (**E**) LH HFD severe liver injury (HE staining, ×200 magnification), and (**F**) LH HFD severe liver injury (Sirius red staining, ×400 magnification).

Severe hepatic injury was observed in LH mice fed the HFD (LH HFD) for 8 weeks but not in LH mice fed control diet (LH CD) as previously reported (Fig. 1A)^21^. Also, WT mice fed the HFD (WT HFD) had higher serum alanine amino transferase (ALT) levels than WT mice fed the CD (WT CD) (Fig. 1A). Macroscopically, yellow discoloration of the liver in WT HFD mice suggested fat deposition (Fig. 1B). However, in the LH HFD mice, the liver surface was irregular and blood congestion was observed, suggesting the existence of a severe inflammatory response in the liver. Liver histopathology revealed that LH CD mice had no hepatic steatosis or inflammation (Fig. 2C). However, in LH HFD mice, not only hepatic steatosis but also inflammatory cell infiltration was observed in portal area (Fig. 2D). Based on the human NAFLD score (NAS), this experimental model showed >66% steatosis (score 3), prominent ballooning (score 2), and 2–4 foci of inflammation per 200x field (score 2), for a total score of 7 (Table 1). In the severe case, aggressive inflammatory cell infiltration and profound necrosis were found in peri-portal and mid-zonal liver parenchyma (Fig. 2E). Although the steatosis seemed diminished in the severe case due to the aggressive parenchymal necrosis, steatosis and ballooning of hepatocytes were able to be confirmed around centri-lobular area of hepatic parenchyma. Based on the NAS score, there was 33–66% steatosis (score 2), prominent ballooning (score 2), and >4 foci of inflammation (score 3), yielding a total score of 7 (Table 1). Sirius Red staining revealed the development of liver fibrosis in necrotic areas (Fig. 2F). In WT mice, HFD caused profound steatosis but no inflammatory cell infiltration or necrosis (Fig. 2B). There was >66% steatosis (score 3), prominent ballooning (score 2), and <2 foci of inflammation, yielding a total score of 5 (Table 1). WT CD mice, WT HFD mice and LH CD mice showed no fibrosis as shown by Sirius Red staining (Suppl. Fig. 1).

### Characteristic F4/80+ macrophages accumulate in HFD fed LH mouse livers

**Figure 3:**
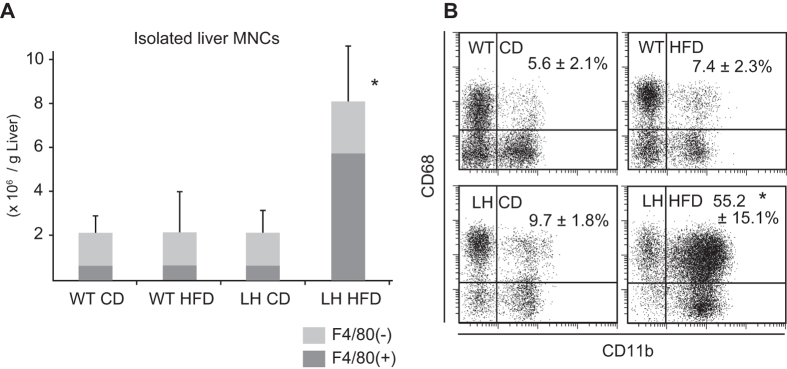
Total counts of liver MNCs and phenotypical characterization of F4/80+ cells. (**A**) Comparison of total MNC counts of each mouse group. Content of F4/80+ cells is represented by dense gray area in the same column. (**B**) Gated F4/80+ cells were examined for their CD11b and CD68 expressions. Liver MNCs were collected by collagenase digestion and gradient centrifugation technique. Isolated MNCs were counted and stained with fluorescent mAbs for flow-cytometry. Data are the means ± SE from 6-8 mice in each group. *p < 0.05 vs. other groups.

The number of isolated hepatic mononuclear cells (MNCs) from HFD LH mice was markedly increased (Fig. 3A), and the proportion of F4/80+ cells was particularly elevated at 8 weeks of HFD (Fig. 3A). These F4/80+ cells in the livers of LH HFD mice primarily consisted of CD11b and CD68 double positive (DP) Kupffer cells/Mφs (Fig. 3B, right lower panel), which were scarce among the liver MNCs from mice in other groups (Fig. 3B). Relatively large FS (forward scatter) and SS (side scatter) measurements (not shown) for these cells, suggested their activated states.

### CD11b+ CD68+ DP Kupffer cells/Mφs produce TNF

**Figure 4:**
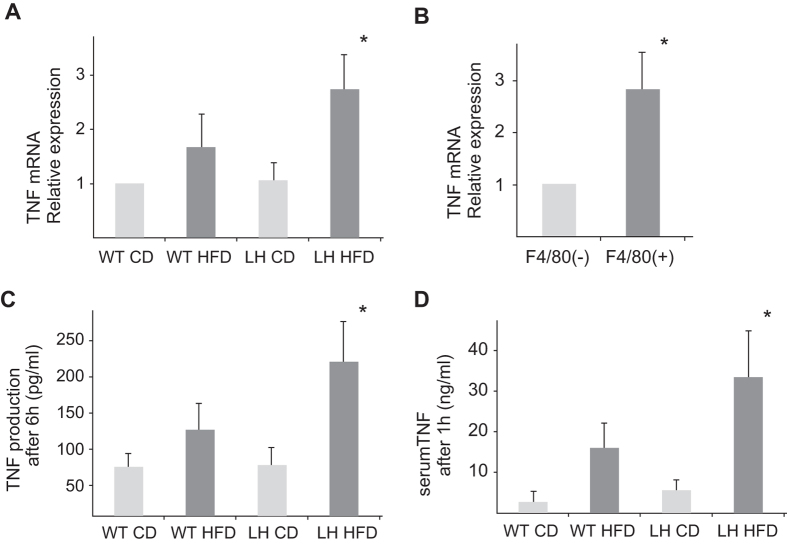
Assessment of TNF production from liver MNCs. (**A**) TNF mRNA expressions of liver MNCs from mice of each group. Data are means ± SE from 5 independent experiments. (**B**) TNF mRNA expressions of MACS-sorted liver F4/80+ cells or F4/80(−) cells. Data are means ± SE from 4 independent experiments. (**C**) *In vitro* TNF production from liver MNCs. Liver MNCs were seeded into microplates and incubated with LPS. The supernatants were collected after 6 h, and TNF concentrations were measured. Data are means ± SE from 4 independent experiments. (**D**) Serum TNF levels after LPS injection. One hour after the intravenous LPS administration, sera from mice of each group were collected and TNF concentrations were measured. Data are means ± SE from 5 mice of each group. *p < 0.05 vs. other groups.

TNF mRNA expression was up-regulated in liver MNCs obtained from LH HFD mice (Fig. 4A). MACS-sorted F4/80+ cells had a much larger amount of TNF mRNA than F4/80(−) cells (Fig. 4B). *In vitro* TNF production from liver MNCs stimulated with LPS was two-fold increased in LH HFD mice (Fig. 4C). TNF production by MACS sorted liver CD11b+ cells obtained from LH HFD mice was significantly larger than that from liver CD11b+ cells from LH CD mice (Suppl. Fig. 2). In contrast, CD11b(−) cells harvested from LH CD and LH HFD mice did not secret TNF. These results indicated that F4/80+ CD11b+ cells in LH HFD mice (most of which are CD68+) are the primary source of TNF. After intravenous LPS injection, LH HFD mice had higher serum TNF levels than WT HFD mice which was compatible with *in vitro* results (Fig. 4D).

### Neutralization of TNF blunts FasL expression of CD11b+ CD68+ DP Kupffer cells/Mφs and inhibits liver inflammation

**Figure 5:**
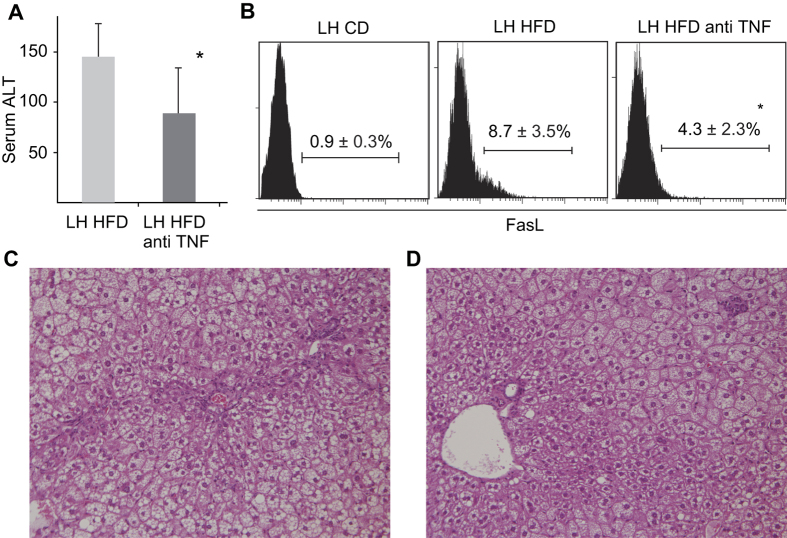
Effect of anti TNF antibody administration on liver injury and FasL expression. FGF5 null LH mice were fed HFD for 8 weeks with or without anti-TNF antibody administration. Sera and livers were collected. (**A**) Serum ALT levels. (**B**) Liver MNCs from mice of each group were stained with fluorescent mAb to FasL, and FasL expressions of gated F4/80+ CD11b+ population were demonstrated. Data are the means ± SE from 5 mice in each group. *p < 0.05 vs. other groups. HE staining of liver in (**C**) normal saline or (**D**) anti TNF antibody injected mice. ×200 magnification.

Anti-TNF antibodies were injected intraperitoneally once a week throughout the 8 weeks of HFD consumption. Serum ALT and liver histopathology were compared with control mice. Serum ALT levels were significantly decreased (Fig. 5A) and liver necrotic areas almost disappeared (Fig. 5C,D). However, the liver steatosis was not attenuated. Surface FasL was expressed in F4/80+ CD11b+ cells from LH HFD (Fig. 5B), and triple staining revealed that these cells (but not other cells including NKT cells) were the dominant population of FasL expression (Suppl. Fig. 3). In WT mice, FasL was not induced by HFD (Suppl. Fig. 4). FasL expression on CD11b+ Kupffer cells/Mφs was significantly blunted by anti TNF antibody treatment (Fig. 5B), which strongly suggests that these cells and FasL are the final effector of this chronic hepatitis model.

### CD11b+ CD68+ DP Kupffer cells/Mφs are recruited Mφs

**Figure 6:**
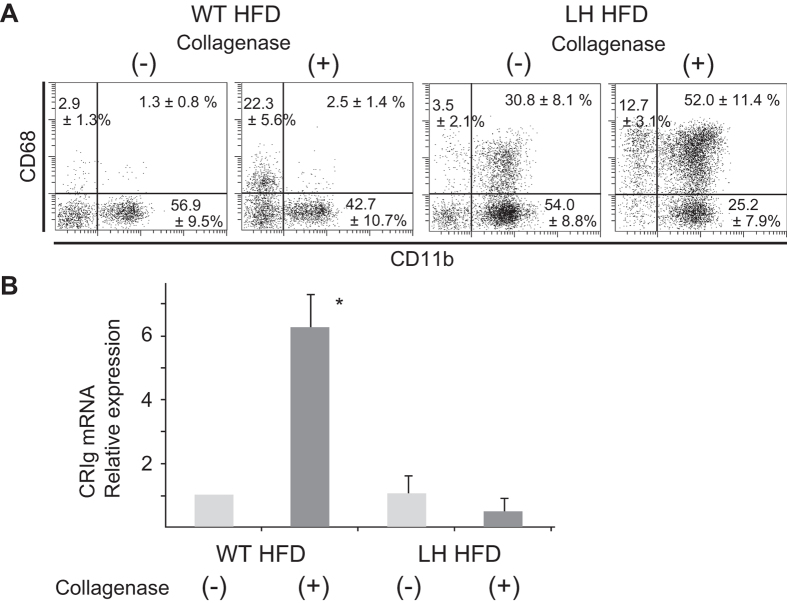
Evaluation of CRIg mRNA of liver MNCs with or without collagenase treatment. (**A**) Liver Kupffer cells/Mφs in mice of each group. (**B**) Quantitative real time PCR analysis of CRIg mRNA in each liver MNC group. Liver MNCs were isolated from LH HFD mice and WT HFD mice with or without collagenase digestion. Data are means ± SE from 4 independent experiments. *p < 0.05 vs. other groups.

CD68+ CD11b(−) resident Kupffer cells were obtained effectively with collagenase treatment in WT HFD mice and LH HFD mice (Fig. 6A), and CRIg mRNA expression was most evident in collagenase treated MNCs from WT HFD mice (Fig. 6B). Although DP Kupffer cells/Mφs could be isolated more effectively in LH HFD mice with collagenase treatment (Fig. 6A), CRIg expression was hardly detected (Fig. 6B), indicating that DP Kupffer cells/Mφs originate from recruited CD11b+ Mφs but not from resident Kupffer cells^12^,^13^.

### NKT cells do not play essential role in this experimental model

**Figure 7:**
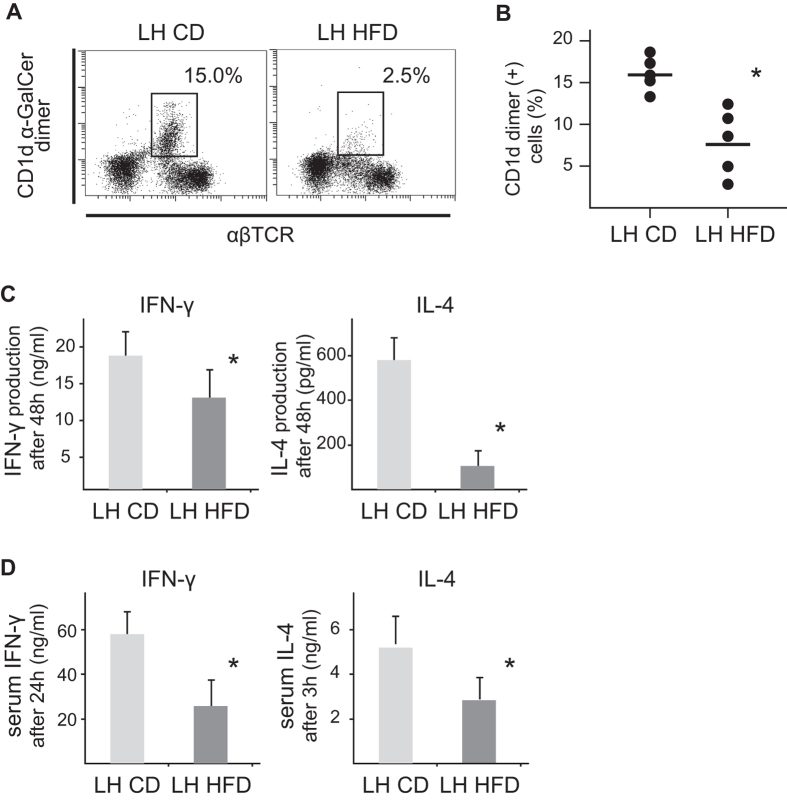
Decrease in NKT cells in the liver, and cytokine production from liver MNCs stimulated with α-GalCer and serum cytokine levels after α-GalCer injection in LH HFD and CD mice. (**A**) Representative flow cytometric analysis of liver MNCs (lymphocytes gated) obtained without collagenase treatment. (**B**) Proportions of NKT cells (%) in liver MNCs (lymphocytes gated). (**C**) IFN-γ and IL-4 production from liver MNCs stimulated with α-GalCer for 48 h *in vitro*. (**D**) Serum IFN-γ and IL-4 levels after α-GalCer injection. Data are means ± SE from 5 mice of each group. *p < 0.05 vs. other groups.

NKT cells significantly decreased in LH HFD mice (Fig. 7A,B). In addition, the cytokine producing function of NKT cells activated by α-GalCer, especially IL-4 production, was impaired (Fig. 7C). Consistent with this, serum IFN-γ and IL-4 levels of mice after α-GalCer injection were reduced (Fig. 7D). These results suggest that the function of NKT cells in FGF5 null mice was impaired by the HFD.

### Intermittent low dose radiation ameliorates liver inflammation and markedly decreases DP Kupffer cells/Mφs

**Figure 8:**
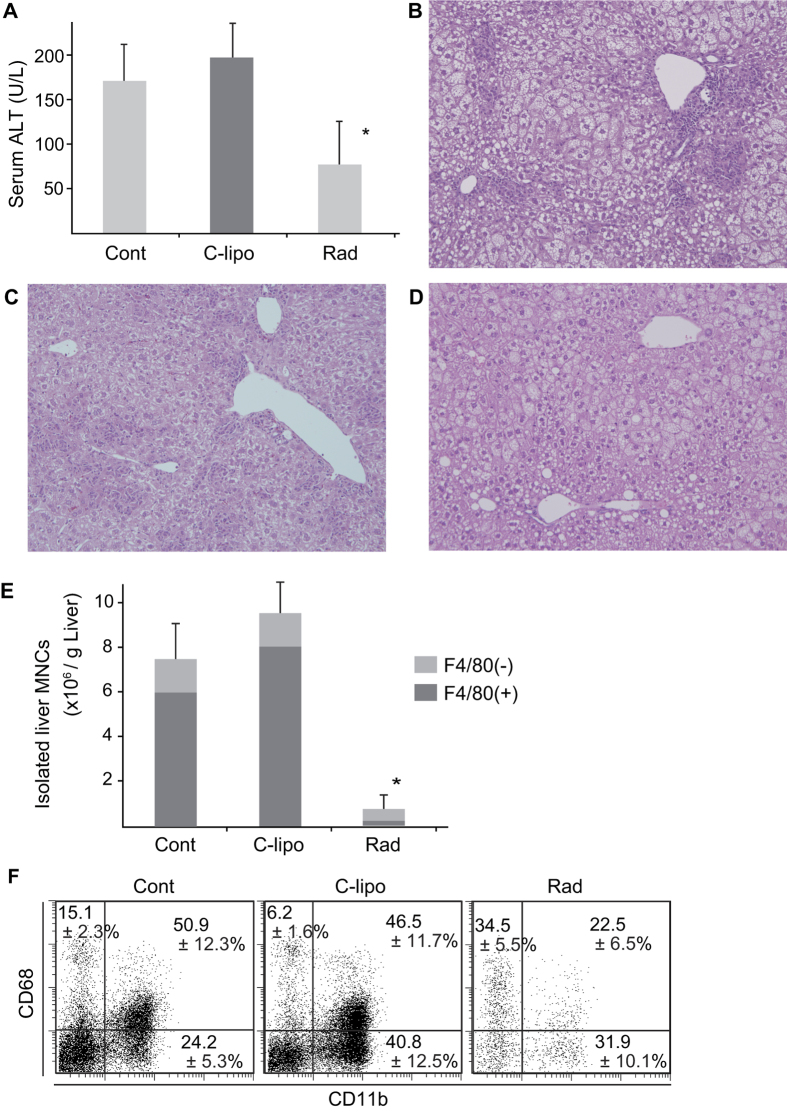
Effect of c-lipo and irradiation on disease progression. (**A**) Serum ALT levels after 8 weeks of HFD in untreated, c-lipo treated (once a week), irradiated (1Gy, once a week) LH HFD mice. Data are means ± SE from 5 mice of each group. *p < 0.05 vs. other groups. HE staining of liver in (**B**) untreated, (**C**) c-lipo treated, (**D**) irradiated mice. ×200 magnification. (**E**) Comparison of total MNC counts in livers of mice in each group. *p < 0.05 vs. other groups. (**F**) Gated F4/80+ cells were examined for their CD11b and CD68 expressions.

LH HFD mice were injected with c-lipo intravenously or irradiated (1 Gy) once a week for eight weeks and serum ALT levels and histopathology findings were compared. Serum ALT was remarkably suppressed by low dose irradiation (Fig. 8A). Pathological examination revealed that although inflammatory cell infiltration was observed in untreated LH HFD mice (Fig. 8B), it was markedly inhibited in irradiated LH HFD mice (Fig. 8D). Furthermore, depletion of resident Kupffer cells by c-lipo tended to increase serum ALT levels and liver inflammation (Fig. 8A,C). However, steatosis of hepatic parenchymal cells was not suppressed by irradiation, resembling simple fatty liver (Fig. 8D).

Low dose irradiation depressed the total liver MNC count, particularly the F4/80+ cell count, while c-lipo administration increased DP Kupffer cells/Mφs accumulation (Fig. 8E). Triple staining revealed that DP Kupffer cells/Mφs were effectively eliminated by irradiation (Fig. 8F), whereas CD68+ resident Kupffer cells were proportionally increased (Fig. 8F). Remaining F4/80 (−) cells include neutrophils (25%), residual NKT cells, NK cells and CD68+ cells (presumably primitive Mφs) and others. T cells and B cells had almost disappeared by 4 weeks (data not shown).

## Discussion

Liver steatohepatitis associated with inflammation and fibrosis was induced in FGF5 null LH HFD mice, in which characteristic DP Kupffer cells/Mφs were recruited to the liver from bone marrow. The production of TNF and FasL suggests that DP Kupffer cells/Mφs may function as effectors in this NASH model. Radiation exposure, but not c-lipo administration, effectively blocked DP Kupffer cells/Mφs recruitment and ameliorated liver inflammation and fibrosis without affecting the fat deposition in hepatocytes, which substantiated our proposition that recruited DP Kupffer cells/Mφs, but not resident Kupffer cells, were mainly involved in the hepatitis and fibrosis created. In addition, NKT cells were decreased and functionally impaired in LH HFD mice, suggesting that they are not profoundly involved in this chronic hepatitis model.

We previously reported that phenotypically and functionally different Kupffer cells^12^ are present in the murine liver and the development of each population is entirely different^13^. Resident CD68 Kupffer cells may develop from c-kit+ precursor cells in the liver, whereas CD11b Kupffer cells/Mφs originate from bone marrow hematopoietic stem cells and are recruited to the liver^13^. Woltman *et al*. commented that the CD11b+ population is the bone marrow derived recruiting population, and the CD11b(−) population is the resident population^26^. Consistent with this, some researchers have proposed that resident liver Kupffer cells are derived from the yolk sac independently from bone marrow hematopoietic stem cells^10^,^11^,^27^,^28^. The functions of these two types of Mφ in the immune system are also quite different. In the case of systemic bacterial infection, resident CD68+ Kupffer cells engulf and kill the bacteria, and CD11b+ Kupffer cells/Mφs are recruited to the liver via the MCP-1/CCR2 axis; CD68+ resident Kupffer cells produce MCP-1 and CD11b+ Kupffer cells/Mφs express CCR2/MCP-1 receptors and are recruited to the liver^13^. The recruited CD11b+ Kupffer cells/Mφs secrete inflammatory cytokines (TNF, IL-12) and IL-12 stimulates NK cells and NKT cells to kill tumor cells (antitumor immunity)^13^. Therefore, the mutual relationship of these distinct Kupffer cell populations is vital for an effective defense mechanism.

The characteristic DP Kupffer cells/Mφs that appeared in LH HFD mice did not express CRIg, which is a crucial marker for distinguishing the CD68+ resident Kupffer cells from recruited CD11b+ Kupffer cells/Mφs^13^,^29^. CD68+ Kupffer cells specifically express CRIg while recruited CD11b+ Kupffer cells/Mφs do not, indicating that the increased DP Kupffer cells/Mφs in LH HFD mice originated from bone marrow-derived recruited Mφs. Consistently, *in vitro* phagocytic activity of F4/80+ CD11b+ cells was relatively low compared to the F4/80+ CD11b(−) population (Suppl. Fig. 5). Considering our previous report demonstrating that CD11b cells express surface CD68 antigen upon activation with LPS^12^, these DP Kupffer cells/Mφs may be the activated state of CD11b+ Kupffer cells/Mφs. In CCl_4_ induced acute hepatitis, the recruited CD11b+ cells produce not only TNF but also FasL, thereby accelerating hepatocyte apoptosis^30^. As in the present study, neutralization of TNF by antibody treatment inhibited FasL expression and ameliorated the CCl_4_ hepatitis. In addition, their functions are upregulated by dietary consumption of high fat and cholesterol diet^6^. These previous results and the present study suggest that DP Kupffer cells/Mφs may have functioned as effectors of this NASH model in FGF5 null LH mice. Additionally, DP Kupffer cells/Mφs expressed not only TNF but also TGF-β mRNA (Suppl. Fig. 6). Considering the previous report that TGF-β expressing CD11b+ recruited Mφs accelerate hepatic fibrosis in CCl_4_ induced liver cirrhosis^4^, DP Kupffer cells/Mφs may be associated with hepatic fibrosis and repair (M2 Mφs)^31^,^32^ in this model.

Bone marrow derived recruited Mφs play a crucial role in atherosclerosis^33^, and obesity and diabetes^34^, by induction of chronic inflammation^35^. In human NASH, recruited CD11b+ Mφs are reportedly detected in liver biopsy specimens, suggesting that the recruitment and activation of these Mφs are also a key factor in the etiology of human NASH^14^,^15^,^16^. Recruited Mφs aggregate and surround fat laden hepatocytes, constituting a hepatic crown like structure in human NASH and experimental models^16^. Interestingly, these Mφs and crown like structure are not eliminated by clodronate liposome treatment^14^, which is consistent with our current findings. In addition, the capacity of CD68+ resident Kupffer cells to secrete chemokines raises the possibility that resident Kupffer cells are the main recruiters of CD11b+ Kupffer cells/Mφs and participate in inflammation and steatohepatitis^13^,^36^,^37^,^38^. There have been two studies demonstrating that c-lipo administration ameliorates liver injury in an experimental NASH model induced by a methionine choline deficient (MCD) diet. in which elimination of resident Kupffer cells by c-lipo ameliorated the steatosis and liver injury by inhibiting MCP-1 and TNF production^37^,^39^. Considering their results, resident Kupffer cells play a certain role in the pathogenesis of steatohepatitis in the MCD diet model. However, in our current model, recruited Mφs, rather than resident population, might be the main effectors in steatohepatitis. Nevertheless, it should be noted that resident Kupffer cells may participate in energy metabolisms such as glucose^40^, cholesterol^41^ and triglyceride^42^. In addition, resident Kupffer cells may produce reactive oxygen species^12^ and play pivotal role as main effectors in Concanavalin A-induced hepatitis^43^. Therefore, the possibility is raised that the depletion of resident Kupffer cells may ameliorate the steatosis and inflammatory response in some liver disease models including MCD diet model. In our recent study, intake of dietary resveratrol, a polyphenol, increased the number and function (uptake of lipids) of CD68+ resident Kupffer cells which in turn markedly suppressed the steatosis of hepatocytes in HFD mice^42^, whereas resveratrol decreased the number of TNF producing CD11b+ Mφs in the liver^42^. The two distinct types of Mφ populations may thus play distinct roles and may affect each other not only with regard to host defense but also to the onset, progress and mitigation of metabolic diseases.

FGF5 gene variance is reportedly associated with hypertension in humans, whereas a role of this gene in lipid/cholesterol metabolism has not been reported. However, as we previously reported, total cholesterol as well as non-HDL cholesterol levels increased in FGF5 null HFD mice^21^, suggesting that LDL cholesterol likely increased as well. Therefore, hypertension in humans with FGF5 variance^22^,^23^,^24^,^25^ should be considered from the viewpoint of lipid/cholesterol metabolism.

Although various types of experimental hepatitis models have been proposed so far, a therapeutic effect of low dose irradiation has not been reported. Compared to a high dose exposure protocol (3 to 4 Gy), intermittent low dose radiation (1Gy) exposure did not cause serious adverse effects such as weight loss and enteritis. These observations raise the possibility that low dose radiation therapy can be a potential therapeutic tool for uncontrolled refractory human NASH patients, in whom it is induced by an excessive inflammatory reaction due to CD11b+ recruited Kupffer cells/Mφs^13^. However, although the liver (hepatocytes) itself is not such a radio-sensitive organ^44^, we cannot exclude the possibility that low dose radiation therapy may increase the susceptibility to hepatocellular carcinoma in NASH patients because of depletion CD11b+ recruited Kupffer cells/Mφs and resultant inactivation of NK cells and NK-T cells.

We previously reported that in chronic hepatitis C patients, CD56+ NK-T cells gradually decreased in proportion and function (cytokine production and tumor killing activity) as the hepatitis progresses to cirrhosis^45^ presumably because intact hepatocytes and their interaction with NK-T cells are required for maintenance of NK-T cells in the liver^45^. These findings in the human liver are consistent with the mouse results in the current study, in which hepatic fibrosis was associated with a reduced number and function of NKT cells. Therefore, we propose that although NKT cells may initiate a Th1 immune response in induction of NASH by the TNF produced by CD11b+ recruited Kupffer cells/Mφs in HFD mice, major effectors in this study could be recruited CD11b+ Kupffer cells, and NKT cells may gradually decrease as a result of hepatocyte damage, as in the livers of hepatitis C patients. Several other studies have suggested that NKT cells may improve chronic hepatic injury because they were decreased in proportion and number in livers in NASH models. However, recent studies have suggested that although NKT cells decreased in mice fed an HFD or in the early stage of a NASH model^3^,^46^, they later increased in number and may play an important role in inducing severe NASH^47^,^48^. These issues should be further addressed in future studies.

Taken together, the present results demonstrate that radio-sensitive CD11b+ Kupffer cells produce TNF and FasL and induce NASH in FGF5 null mice, which is effectively attenuated by low dose radiation therapy.

## Methods

### Mice and diets

The Ethics Committee of Animal Care and Experimentation, National Defense Medical College, Japan, approved all requests for animals and the intended procedures of the present study (Permission number: 15025). All experiments were performed in accordance with relevant guidelines and regulations. Spontaneous FGF5 null LH mice (ICR background, males) were bred and maintained in our laboratory. After becoming 4 weeks old, they were fed the HFD (TD.88137, Harlan Laboratories, Madison,WI, USA) or CD (NMF, ORIENTAL EAST, Tokyo, Japan) for 8 weeks. WT male ICR mice (4 weeks of age) were purchased from Japan SLC (Hamamatsu, Japan) and fed the HFD or CD in a similar manner. The HFD contained 21.2% fat, 0.2% cholesterol, 17.3% protein, 48.5% carbohydrate, and 4.5 kcal/g. The CD contained 3.8% fat, 17.7% protein, 59.4% carbohydrate, and 3.4 kcal/g. The mice had unlimited access to water and food during the study period.

### Isolation of mononuclear cells, including Kupffer cells

The murine livers were removed under deep anesthesia. The liver MNCs were prepared essentially as described previously. Briefly, the livers were minced and suspended in HBSS containing 0.05% collagenase (Wako, Osaka, Japan), and then were shaken for 20 min in a 37 °C water bath. Next, the liver specimens were filtered through a stainless steel mesh and remnants were dissolved using a rubber stick on the mesh. After mixing in isotonic 33% percoll solution containing heparin, the samples were centrifuged for 20 min at 850 × g at room temperature. After removing the supernatant, the pellets were resuspended in a red blood cell lysis solution and then were washed twice in 1% FBS RPMI 1640. For some experiments, liver MNCs without resident CD68+ CD11b(−) Kupffer cells were obtained by not treating liver specimens with collagenase.

### Measurement of serum alanine aminotransferase, and cytokine levels

Blood samples were taken from the inferior vena cava when the liver specimens were collected. The serum ALT level was measured using a DRICHEM 3000V instrument (Fuji Medical Systems, Tokyo, Japan). The serum and *in vitro* supernatant TNF levels were measured by ELISA kits for TNF (Thermo Fisher Scientific, Waltham, MA, USA).

### Pathological examination

For the pathological examination, the removed livers were immersed in 10% formalin for two days. Slides were prepared from them and stained with hematoxylin and eosin (HE) in our laboratory. For assessment of fibrosis, Sirius red staining was performed (MorphoTechnology, Sapporo, Japan).

### Flow cytometry

The MNCs were incubated for 15 min at 4 °C with Fc-blocker (2.4 G2; BD PharMingen, USA) to prevent any nonspecific binding. For identification of Kupffer cells, MNCs were stained with a FITC labeled anti-F4/80 antibody (Ab) (BM8, eBioscience, San Diego, CA. USA), PE-Cy5 labeled CD11b Ab (M1/70, BD PharMingen), and biotin labeled CD68 Ab (FA-11, SEROTEC, Oxford, UK) and developed with PE-streptavidin (eBioscience). For analysis of FasL expression, a PE labeled FasL antibody (Kay-10, eBioscience) was used. Flow cytometric analysis was performed using a Cytomics FC500 instrument (Beckman Coulter, Indianapolis, IN, USA).

### Isolation of F4/80+ CD11b+ Kupffer cells from LH mice using MACS sort system

The isolated liver MNCs were treated with Fc-blocker as mentioned above, and stained with a PE-Cy5 labeled anti-F4/80 antibody following conjugation with anti-PE magnetic beads (Miltenyi Biotec, Bergisch Gladbach, Germany). F4/80+ cells conjugated with magnetic beads or unconjugated F4/80(−) cells were sorted by Super MACS system (Miltenyi Biotec).

### Real-time gene expression analysis of liver MNCs

RNeasy Mini Kit (Qiagen, Hilden, Germany) was used to isolate mRNA from about 2.0 million harvested liver MNCs. Complementary DNA was generated from 500 ng RNA using a SuperScript III First-Strand Synthesis System (Life technologies, Carlsbad, CA, USA). A quantitative real-time polymerase chain reaction (PCR) was performed using FastStart SYBR Green Master Reagent and Light cycler 480 System (Roche Applied Science, Penzberg, Germany). Reactions were conducted twice in triplicate and Rsps18 values were used to normalize gene expression. For mouse TNF, antisense primer (TCACCCCGAAGTTCAGTAGACA) and sense primer (CCAGAAAAGACACCATGAGCAC) were used; for mouse Rps18, antisense (CCAGTGGTCTTGGTGTGCTGA) and sense (TTCTGGCCAACGGTCTAGACAAC); and for mouse TGF-β, antisense (CGTTGATTTCCACGTGGAGT) and sense (CAACAATTCCTGGCGTTACC) were used.

### Preparation and use of reagents

50 mg of clodronate (LKT Laboratories Inc, St. Paul, MN, USA) was dissolved in 500 μl water and mixed with COATSOME EL-01-A (Nichiyu, Tokyo, Japan) to encapsulate it into c-lipo. The prepared c-lipo was diluted with 7.5 ml normal saline and 200 μL was injected into 20 g mice via the tail vein. Depletion of CD68+ Kupffer cells was confirmed in a period of about 1 week. The c-lipo was administered one day before feeding with the HDF or CD, and then once a week for 8 weeks. The dietary intake of mice administered with it was confirmed to be no different from that of the control mice. Mice were i.v. challenged with 2.5 mg/kg body weight *Escherichia coli*- LPS (SIGMA, St. Louis, US) or 100 μg/kg α-GalCer (Funakoshi Co. Ltd., Tokyo, Japan)^49^. In *in vitro* experiments, 5 × 10^5^ liver MNCs/200 μl were stimulated with LPS (10 μg/ml) or α-GalCer (100 ng/ml). Neutralization of TNF was performed by intermittent i.p. administration of 250 μg anti TNF antibody (MP6-XT3; IBL bioscience, Gunma Japan) to LH mice once a week throughout the HFD consumption.

### Intermittent low dose radiation exposure

Radiation exposure was begun one day before the HFD or CD. To prevent adverse effects, the radiation dose was limited to 1 Gy and exposure was once a week for 8 weeks. The dietary intake of the irradiated mice was confirmed to be not different from that of the control mice and gastrointestinal symptoms were not observed.

### Statistical analysis

Data are presented as mean values ± SE. The statistical analyses were performed using a JMP pro 12 software package (SAS Software, Cary, NC, USA). For the comparison of two groups, the two tailed student T test was performed. For the multiple comparisons of more than 3 groups, standard one-way analysis of variance was performed, followed by the Tukey-Kramer’s test. P < 0.05 was considered to indicate a significant difference.

## Additional Information

**How to cite this article**: Nakashima, H. *et al*. Activation and increase of radio-sensitive CD11b+ recruited Kupffer cells/macrophages in diet-induced steatohepatitis in FGF5 deficient mice. *Sci. Rep.* **6**, 34466; doi: 10.1038/srep34466 (2016).

## Supplementary Material

Supplementary Information

## Figures and Tables

**Table 1:** NAS score of each liver specimen.

	Steatosis	Balloonig	Inflammation	Total
WT CD	0	0	0	0
WT HFD	3	2	0	5
LH CD	0	0	0	0
LH HFD moderate	3	2	2	7
LHHFD severe	2	2	3	7
