# Supplementary material for: Activation and increase of radio-sensitive CD11b+ recruited Kupffer cells/macrophages in diet-induced steatohepatitis in FGF5 deficient mice
Source: Sci Rep. 2016 Oct 6;6:34466. doi: 10.1038/srep34466 (PMC5052649; doi:10.1038/srep34466)
Supplement: Supplementary Information [file srep34466-s1.doc]

**Activation and increase of radio-sensitive CD11b+ recruited Kupffer cells/macrophages in diet-induced steatohepatitis in FGF5 deficient mice**

Hiroyuki Nakashima^1^, Masahiro Nakashima^1^, Manabu Kinoshita^1^,

Masami Ikarashi^1^, Hiromi Miyazaki^3^, Hiromi Hanaka^2^, Junko Imaki^2^, and Shuhji Seki^1^

^1^Department of Immunology and Microbiology

^2^Department of Developmental Anatomy and Regenerative Biology

National Defense Medical College, Namiki 3-2, Tokorozawa, Saitama 359-8513, Japan

^3^Department of Traumatology

National Defense Medical College Research Institute, Namiki 3-2, Tokorozawa, Saitama 359-8513, Japan

**
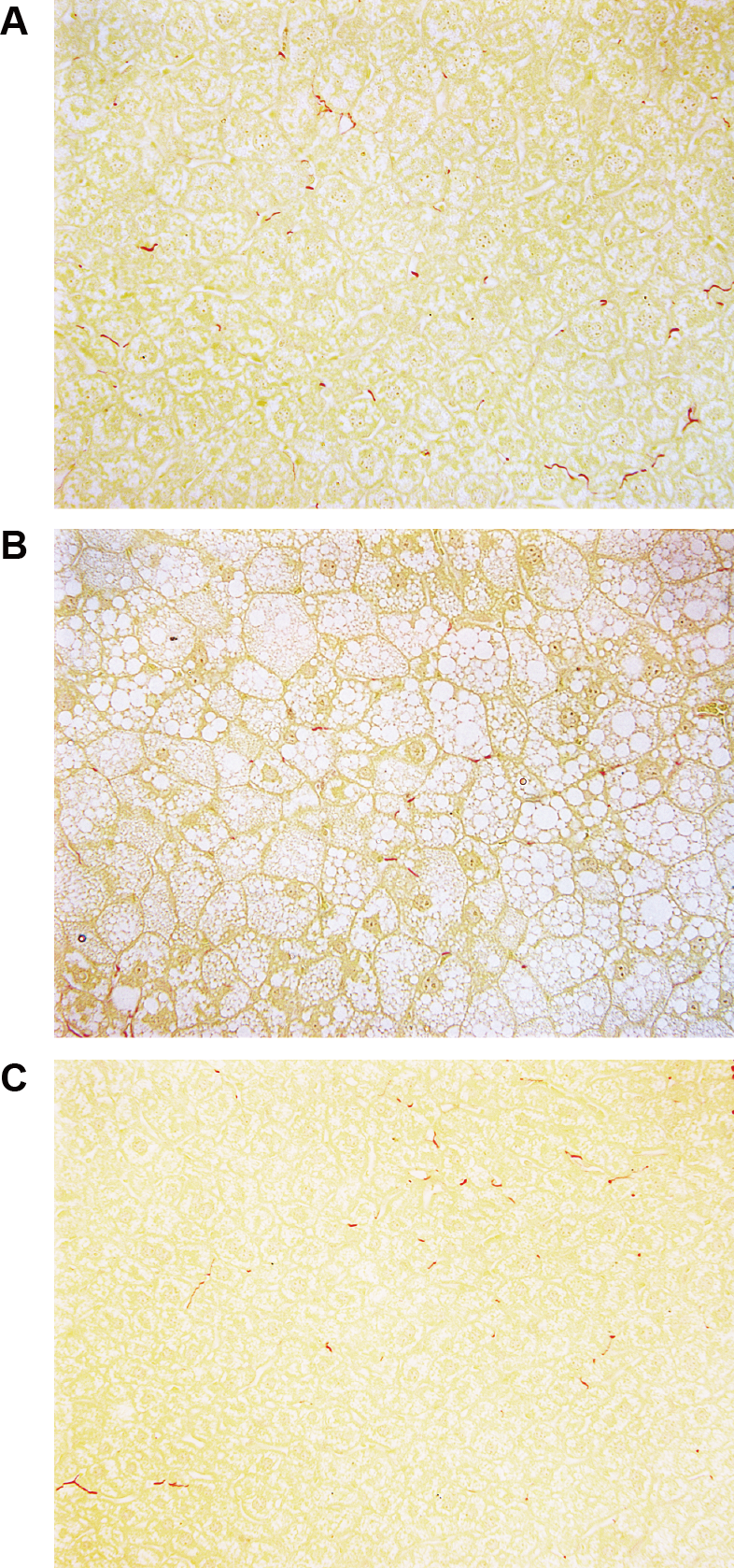
**

**Supplementary Figure 1. Sirius red staining.**

(A) WT CD, (B) WT HFD, (C) LH CD. ×400 magnification.

**
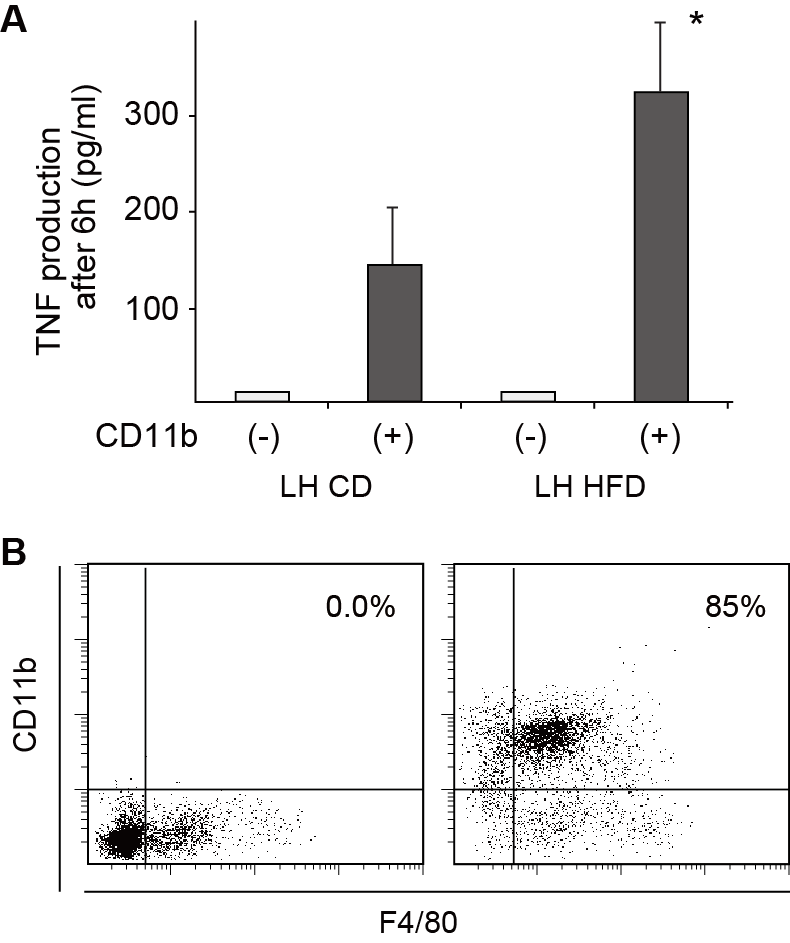
**

**Supplementary Figure 2. TNF production of MACS sorted CD11b (-) or (+) cells from LH CD or HFD mice.**

Liver MNCs were isolated from LH CD and HFD mice with collagenase digestion. CD11b (+) or (-) cells were sorted with Super MACS system. (A) Sorted MNCs were incubated with LPS and TNF productions in culture supernatants were evaluated. (B) Sorted CD11b (+) or (-) MNCs from LH HFD mice were stained with F4/80 antibodies and the purity of each type of cell analyzed.

**
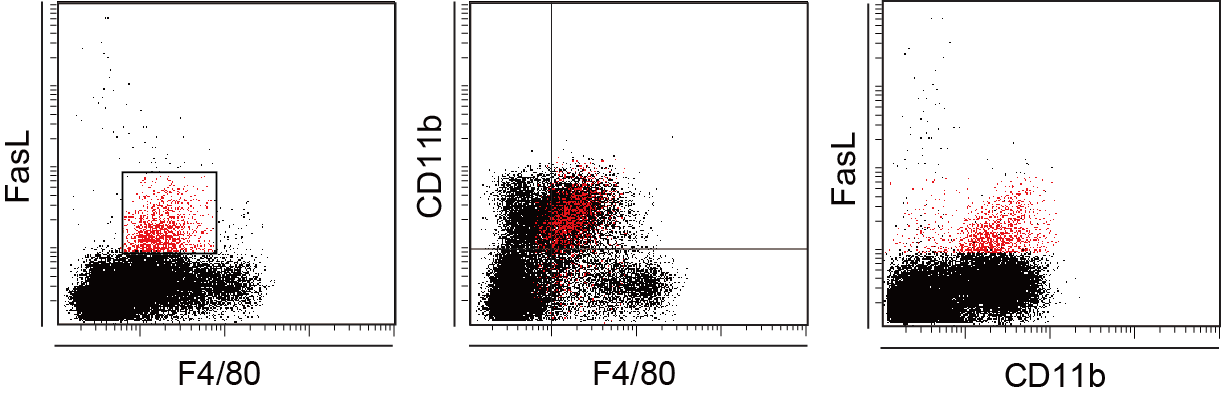
**

**Supplementary Figure 3. FasL expression of liver CD11b+ Kupffer cells/Mφs in LH HFD mice**

Liver MNCs were isolated from LH HFD mice, and stained with F4/80, FasL, and CD11b. FasL was mainly expressed in F4/80+ population (Left panel), and gated FasL+ cells, which were indicated with red dots, were included in the F4/80 and CD11b double positive area. This finding demonstrates that the CD11b+ Kupffer cells/Mφs are the most dominant population of FasL expression.

**
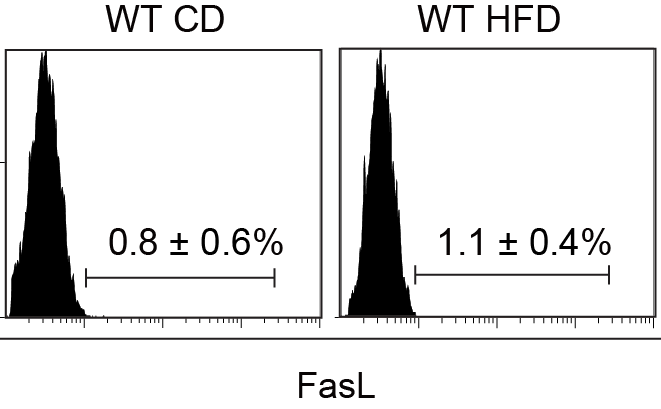
**

**Supplementary Figure 4. FasL expression of liver CD11b+ Kupffer cells/Mφs in wild type mice**

Wild type ICR mice were fed CD or HFD for 8 weeks and liver MNCs were collected and FACS analysis was performed to analyze FasL expression. FasL was not expressed on CD11b+ Kupffer cells/Mφs in either CD or HFD fed mice. Data are means ± SE from 4 mice of each group. * *p* < 0.05 vs. other groups.

**
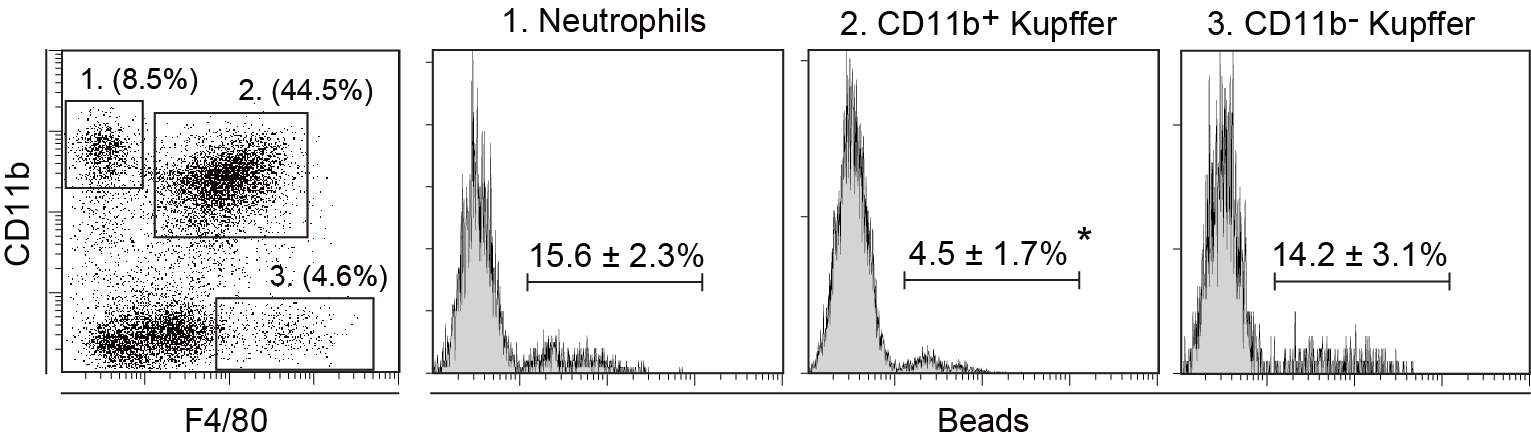
**

**Supplementary Figure 5. Evaluation of phagocytic activity of liver Kupffer cells/Mφs and neutrophils**

Liver MNCs isolated from LH HFD mice were seeded into microplates and incubated with fluorescent microbeads. Liver MNCs and neutrophils were collected after 30 min of incubation, then stained with F4/80 mAb and CD11b mAb, and analyzed by flow cytometry. Data are means ± SE from 4 mice of each group. * *p* < 0.05 vs. other groups.


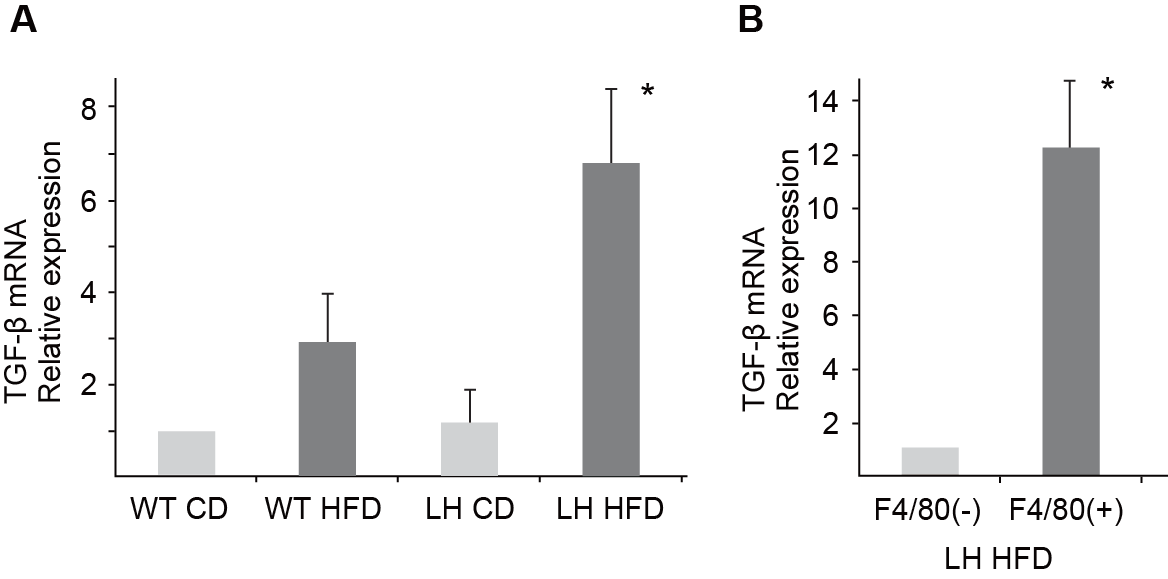


**Supplementary Figure 6. CD11b+ CD68+ DP Kupffer cells/Mφs** **produce TGF-β**

(A) TGF-β mRNA was markedly up-regulated in LH HFD mice. (B) Also, mRNA analysis of MACS sorted F4/80+ cells from LH HFD mice revealed that the increased DP Kupffer cells/Mφs were the primary source of TGF-β, suggesting that DP Kupffer cells/Mφs may be involved in hepatic fibrosis. Results from PCR are expressed as fold induction compared to CD fed WT mice. Data are means ± SE from 4 independent experiments. * *p* < 0.05 vs. other groups.
